# Effects of mixed meal tolerance test on gastric emptying, glucose and lipid homeostasis in obese nonhuman primates

**DOI:** 10.1038/s41598-021-91027-3

**Published:** 2021-06-04

**Authors:** Kamal Albarazanji, Andrea R. Nawrocki, Bin Gao, Xiaoli Wang, Yixin (Jim) Wang, Yong-Fu Xiao

**Affiliations:** 1 Janssen Research & Development, Cardiovascular and Metabolism, 1400 McKean Rd., Spring House, PA 19477 USA; 2 Cardiovascular and Metabolic Diseases, Crown Bioscience, Inc., 6 Beijing West Road, Taicang, Jiangsu Province 215400 People’s Republic of China

**Keywords:** Gastrointestinal hormones, Metabolism, Metabolic syndrome

## Abstract

Meal ingestion elicits a variety of neuronal, physiological and hormonal responses that differ in healthy, obese or diabetic individuals. The mixed meal tolerance test (MMTT) is a well-established method to evaluate pancreatic β-cell reserve and glucose homeostasis in both preclinical and clinical research in response to calorically defined meal. Nonhuman primates (NHPs) are highly valuable for diabetic research as they can naturally develop type 2 diabetes mellitus (T2DM) in a way similar to the onset and progression of human T2DM. The purpose of this study was to investigate the reproducibility and effects of a MMTT containing acetaminophen on plasma glucose, insulin, C-peptide, incretin hormones, lipids, acetaminophen appearance (a surrogate marker for gastric emptying) in 16 conscious obese cynomolgus monkeys (*Macaca fascicularis*). Plasma insulin, C-peptide, TG, aGLP-1, tGIP, PYY and acetaminophen significantly increased after meal/acetaminophen administration. A subsequent study in 6 animals showed that the changes of plasma glucose, insulin, C-peptide, lipids and acetaminophen were reproducible. There were no significant differences in responses to the MMTT among the obese NHPs with (n = 11) or without (n = 5) hyperglycemia. Our results demonstrate that mixed meal administration induces significant secretion of several incretins which are critical for maintaining glucose homeostasis. In addition, the responses to the MMTTs are reproducible in NHPs, which is important when the MMTT is used for evaluating post-meal glucose homeostasis in research.

## Introduction

Glucose metabolism after a mixed meal is influenced by meal composition, rates of gastric emptying and hormonal responses to nutrient ingestion. The Mixed Meal Tolerance Test (MMTT) requires a subject to drink or to be orally administered with a "mixed meal" (comprised of defined amounts of carbohydrates, fats and proteins) after overnight fast^1^. The purpose of the test is to measure pancreatic β-cell insulin secretion and glucose tolerance in response to the meal-induced increases in digested nutrients. A healthy pancreas releases precise amounts of insulin to normalize blood glucose during the test. Therefore, the MMTT physiologically resembles the postprandial metabolic homeostasis in daily life. In condition with impaired insulin secretion such as in insulin-dependent type 1 and type 2 diabetes mellitus (T2DM), the MMTT is used to assess β-cell function and is regarded as more physiological than the glucagon stimulation test (GST), which is a standard measure of endogenous insulin secretion^2–4^. Glucagon is a potent stimulus for the islet beta-cell and intravenous bolus injection of 1 mg glucagon has been widely used to assess endogenous insulin secretion for clinical and research purposes^3–5^. MMTT-induced stimulation of insulin release involves an enteroinsular axis via incretin hormones, such as gastric inhibitory peptide (GIP), glucagon-like peptide-1 (GLP-1), amongst others. These hormones are primarily produced by enteroendocrine cells of the gut and secreted into the blood stream after meal ingestion. Therefore, the MMTT assesses both the pancreas function as well as the integrity of the enteroinsular axis through GLP-1 and other incretin hormone releases^6–8^. Hence, the MMTT is often used in both clinical and pre-clinical research to assess metabolic homeostasis in diabetes and obesity research^9–12^.

T2DM is a worldwide health issue and is mainly associated with a global increase in prevalence of obesity. Various animal models have been used in research for understanding the diseases and discovering novel therapies^13–16^. Nonhuman primates (NHPs) can naturally become obese and develop T2DM in a manner similar to the onset and progression of T2DM in humans, which makes them excellent models for diabetes and obesity research^15–24^.

Gastric emptying is the process by which the contents of the stomach are moved into the duodenum. The acetaminophen method has been used to determine the gastric emptying rate in monkeys and humans, because the rate of orally administered acetaminophen absorption accurately reflects the rate of gastric emptying^25,26^. It is known that acetaminophen is not absorbed from the stomach but rather from the small intestine. After nasogastric tube administration, acetaminophen is absorbed almost exclusively in the proximal portion of the small intestine. If the small intestine is healthy, the rate of gastric emptying is the rate-limiting step in its absorption^24,27^. Therefore, the kinetics of the appearance of acetaminophen in plasma after single oral administration is considered as an indirect method for determining the rate of gastric emptying^27,28^. Approximately 30%-50% of patients with prolonged diabetes have abnormally slow gastric emptying^29^. The rate of gastric emptying contributes as much as 35% of peak glycemia following a meal^30^.

The present study aimed to assess the effects of MMTT mixed with acetaminophen similar to a MMTT with paracetamol that is used in clinical studies on gastric emptying^31^ on plasma glucose and lipid levels and hormone secretion in obese nonhuman primates (*Macaca fascicularis*) with or without diabetes. Based on our results, we confirm the feasibility and reproducibility of a clinical MMTT protocol in conscious normo- and hyper-glycemic obese NHPs, which can enable testing of drug candidates or address mechanistic questions related to pancreatic and endocrine function in obese and/or diabetic models. In addition, the comprehensive assays of blood incretins and gastric emptying conducted in this study can provide some insights for a clinical MMTT challenge to look at the changes of incretins and gastric movement, especially in obese and diabetic patients. Moreover, the hyperglycemic obese monkeys showed some enhancement in incretins, such as tGIP, during the MMTT challenge, which suggests that under a hyperglycemic obese condition some compensatory upregulation of incretins may help to maintain blood glucose homeostasis during and after a meal ingestion.

## Materials and methods

### Animal selection and animal care

The experiments were performed in male obese cynomolgus monkeys (Table 1) originally obtained from Jinjiekang monkey farm (Yuanjiang, Yunnan Province, China) and raised in our animal facility for 3 to 7 years (Taicang, Jiangsu Province, China). During the study, the enrolled monkeys were also grouped into those who had diabetes or not, based on our previous criteria^17,18,32^. Monkeys were individually housed and maintained in accordance with the guidelines of the Association for Assessment and Accreditation of Laboratory Animal Care (AAALAC). The room temperature was maintained at ∼ 21 °C with a 12 h light/dark cycle with lights off from 7 PM to 7 AM. Animals were allowed ad libitum access to water and a complete, nutritionally balanced normal diet (Beijing Keao Xieli Feed Co., LTD, Beijing, China), enriched with seasonal fruit and vegetables. The experimental protocol was approved by the Institutional Animal Care and Use Committee (IACUC) of Crown Bioscience, Inc.

**Table 1:** General characteristics of the enrolled cynomolgus monkeys.

	1st MMTT (n = 16)	2nd MMTT (n = 6)	*P* value
Age (year)	15.1 ± 0.6	15.1 ± 0.8	0.94
Body weight (kg)	11.2 ± 0.3	11.3 ± 0.5	0.79
Total fat (%)	24.2 ± 7.3	21.8 ± 4.3	0.80
Glucose (mmol/L)	6.1 ± 0.5	6.9 ± 0.8	0.37
Insulin (mIU/L)	106 ± 16	132 ± 19	0.37
C-peptide (nmol/L)	1.9 ± 0.2	2.1 ± 0.3	0.44
Triglyceride (mmol/L)	1.6 ± 0.2	1.5 ± 0.2	0.97
Cholesterol (mmol/L)	2.5 ± 0.1	2.3 ± 0.2	0.44
HDL (mmol/L)	0.9 ± 0.04	0.9 ± 0.1	0.34
LDL (mmol/L)	1.1 ± 0.1	1.0 ± 0.1	0.77

Values are expressed as mean ± SEM. *P* value, versus 1st MMTT group.

Table 1 summarizes the general characteristics of the animals and reproducibility of the measured parameters. Before MMTT, animals were trained once daily for 2 weeks to sit in a monkey chair for at least 60 min while restrained, and body weights were measured after an overnight-fast (approximately 16 h). Blood (2 mL/each) for biochemistry analysis to group the animals was collected from a cephalic or saphenous vein into a K_2_-EDTA tube, gently inverted 10 times, and then immediately placed on ice. Animals were returned to cages after blood collection. Samples were centrifuged at 3000 rpm for 15 min at 4 °C to collect plasma. All samples kept at 4 °C were analyzed within 3 to 4 h after collection. Plasma glucose (by Siemens Advia-2400) and insulin (by Siemens Advia Centaur XP) were analyzed in a clinical lab (The First People’s Hospital of Taicang, Jiangsu Province, China).

Body composition was measured by dual-energy X-ray absorptiometry (DEXA) scan (GE Model: Lunar DPX-NT, Milwaukee, WI, USA)^33^ in overnight fasted (approximately 16 h) anesthetized (ketamine, initial dose of 10 mg/kg i.m., maintained with 5 mg/kg i.m. as needed) animals.

### Preparation of the mixed meal with acetaminophen (4 mg/mL)

Ensure Plus (Abbott Labs, USA; Per 8 oz [237 mL] bottle consists of total fat [11 g], cholesterol [0.01 g], total carbohydrates [51 g containing 1 g dietary fiber and 22 g sugar), protein [13 g] and various vitamins plus minerals) was used for the MMTT as previously reported^34^. Acetaminophen (Sigma-Aldrich, USA) was slowly added to Ensure Plus solution (4 mg/mL) and continuously stirred for 6 h. The prepared meal solution containing acetaminophen was kept at room temperature (22 °C) and protected from light until being used within 24 h. The meal solution was continuously mixed with a magnetic stir-bar at least 30 min before dosing.

### Procedures of the mixed meal tolerance test

The enrolled animals were trained and acclimatized chair restraint and oral gavage via nasogastric tube daily for 2 weeks. Animals were weighed in the morning after an overnight fasting (~ 16 h). At approximately 8:00AM, immediately after baseline blood sampling animals received the mixed meal plus acetaminophen solution via a nasogastric tube (5 mL/kg) followed by a 5 mL flush with water. To prevent hypoglycemia during the late stage of the MMTT because of 16 h overnight fasting plus 5 h MMTT procedure, the animals were intravenously infused slowly over 5 min with 2.5 mL/kg of 20% glucose solution at the end of the procedure (300 min time-point). After completion of the MMTT, animals were returned to their individual cages. No clinical abnormalities were observed during or after the MMTT.

### Blood sampling and handling

Blood (4.8 mL per animal per time point) was collected from the cephalic vein immediately before (0) and then at 15, 30, 60, 90, 120, 180, 300 min after the meal administration. Each animal was chair-restrained until finishing blood collection of 30 min time point and then was returned to its cage. The animal was re-chaired approximately 5 min before the next time point for blood collection and then repeated the procedure until completion of the last time point of blood collection. Plasma samples were analyzed for glucose, insulin, C-peptide, TG, TC, HDL, LDL, several incretins and acetaminophen levels.

Blood was collected into a BD P800 blood collection tube (BD Bio Sciences, USA) coated with K_2_EDTA and protease inhibitors, including dipeptidyl peptidase IV (DPP-IV) inhibitor (D4943, Millipore Sigma, USA). Blood samples were gently inverted 10 times and then centrifuged at 3500 rpm for 10 min at 4 °C. Plasma stored at 4 °C for analysis of glucose, insulin, C-peptide, TG, TC, HDL and LDL was used within 4 h after collection. Other plasma samples for acetaminophen, GLP-1 (active and total), GIP and PYY assays were stored in a freezer at − 80 °C until analysis. Acetaminophen concentrations in the plasma samples were measured according to the method described previously^24,26^.

### Hormone assays

All samples were assayed in duplicate.

Total GLP-1 concentrations in the collected plasma samples were quantified using Meso Scale Discovery Total GLP-1 (ver. 2) Assay Kit (Cat. No. K150JVC, Lot No. K0040378, Rockville, MD, USA) in a sandwich immunoassay method. The lower limit of detection (LLOD) of this kit is 0.98 pg/mL, and the specificity for cynomolgus monkey was validated using a spiked recovery method to determine the minimum required dilution needed for analysis. Six samples of known Total GLP-1 concentrations were tested 2 times per samples on one 96 well plate to assess intra-assay precision. The K150JVC kit was pre-coated with Total GLP-1 capture antibodies and the assay is to detect electrochemiluminescent from native Total GLP-1 in the samples based on Total GLP-1 capture antibody-Total GLP-1 Antigen-Total GLP-1 SULFO-TAG labeled detection antibody interactions (immunosorbency). Quality control assays reproducibility to be identified as the intra-assay CV (%) and inter-assay CV (%).Standards and samples were pipetted into the K150JVC wells and any Total GLP-1 present was bound by the immobilized capture antibody on the working electrode surface. After removing any unbound substances through washing, the MSD SULFO-TAG detection antibody specific for Total GLP-1 was added to the wells which bound to the immobilized Total GLP-1 antigen of the sample. After washing, MSD read buffer was added to the wells which provides the appropriate chemical environment for electrochemiluminescence. The plate was loaded into the MSD instrument for analysis (Meso Scale Sector S600, Serial No. 1201160502430). Within the instrument, a voltage was applied to the plate electrodes which caused the sandwiched immunoassay labels bound to the working electrode surface to emit light. The instrument measures the intensity of the emitted light to quantitate the Total GLP-1 concentrations within the sample. Active GLP-1 (7–36 and 7–27) forms in the collected cynomolgus monkey plasma samples were quantified using Meso Scale Discovery Active GLP-1 (ver. 2) Assay Kit (Cat. No. K150JWC, Lot No. K0040371, Rockville, MD, USA) in a sandwich immunoassay method. The LLOD of this kit is 0.12 pg/mL. The assay procedures were the same or very similar to those detailed above for Total GLP-1 assay and readout was by the same MSD instrument (Meso Scale Sector S600, Serial No. 1201160502430).

Total GIP concentrations in the collected cynomolgus monkey plasma samples were quantified using Meso Scale Discovery Human Total GIP Assay Kit (Cat. No. K151RPD, Lot No. K00E0315, Rockville, MD, USA) in a sandwich immunoassay method. The assay has high sensitivity and excellent specificity for detection of non-human primate Total GIP according to the product description. The LLOD of this kit is 5.0 pg/mL, and the specificity for cynomolgus monkey was validated using a spiked recovery method to determine the minimum required dilution needed for analysis. The assay procedures were the same or very similar to those detailed above for total GLP-1 assay and readout was by the same MSD instrument (Meso Scale Sector S600, Serial No. 1201160502430).

PYY concentrations in the collected cynomolgus monkey plasma samples were quantified using MyBioSource Monkey PYY (Peptide YY) ELISA Kit (Cat. No. MBS2501419, Lot No. AK0018APR02098 and AK0018FEB09043, San Diego, CA, USA) utilizing an enzyme-linked immunosorbent assay method (ELISA). The LLOD of this kit is 18.75 pg/mL, and the specificity for cynomolgus monkey was validated using a spiked recovery method to determine the minimum required dilution needed for analysis. The ELISA kit is designed to detect native, not recombinant, PYY by employing a two-site sandwich immunoassay. A capture antibody specific for PYY has been pre-coated onto a microplate. Standards and samples were pipetted into the wells and any PYY present was bound by the immobilized capture antibody within the micro-well plate. A biotinylated detection antibody specific for monkey PYY and Avidin-Horseradish Peroxidase (HRP) conjugate were added to each well successively and incubated. After removing any unbound substances through washing, the substrate solution was added to the wells. Only wells containing bound PYY, biotinylated detection antibody and HRP conjugate reacted to produce a blue color. Stop solution was added to the enzyme–substrate reaction which then changed the color from blue to yellow. The optical density (OD) was measured spectrophotometrically using the BioTek Synergy 2 Plate Reader (Serial No. 1312121D) at a wavelength of 450 nm and the concentrations of PYY in the samples were calculated based on their OD values by comparing the OD of the samples to the standard curve.

### Data analysis

The area under the curve (AUC) of several parameters, such as acetaminophen, glucose, insulin, C-peptide, TG, GLP-1, tGIP and PYY, was calculated using the first measurement (t = 0 min, baseline) as reference (M1 method) exactly as described previously^23^. To evaluate the degree of insulin resistance, the homeostasis model assessment of insulin resistance (HOMA-IR) was calculated by the equation of fasting plasma glucose concentration (mmol/L) x fasting plasma insulin concentration (mIU/L)/22.5^35,36^. All data are expressed as mean ± standard error of the mean (SEM). Statistical analysis was performed by using two-tailed Welch’s t-test for comparison of two means and by using One Way or Two Way repeated measures with analysis of variance (ANOVA, GraphPad Prism 8.0.1) for multiple means and then by Dunnett’s or Sidak’s multiple comparisons test. A *p*-value less than 0.05 after comparing every mean with the self-baseline mean (at time 0 min as pre-administration of MMTT) or the corresponding set of means from the hypo- and hyper-glycemic monkeys or results of post-hoc analysis was considered statistically significant. A power simulation analysis was performed for acetaminophen using Software R and related package followed by two-sample one sided-test for power-calculation.

### Research involving animals

This study was conducted in nonhuman primates, cynomolgus monkeys. The study protocol and experimental procedures used in this study were approved by the IACUC of Crown Bioscience Inc., which includes member from outside of the company. The approval numbers are AN-1702-009-14 and AN-1702-009-15. The study was also carried out in compliance with the ARRIVE guidelines.

### Informed consent

All the authors have read and approved the manuscript for submission. Their consents are available if requested.

## Results

### Metabolic responses to a MMTT

**Figure 1:**
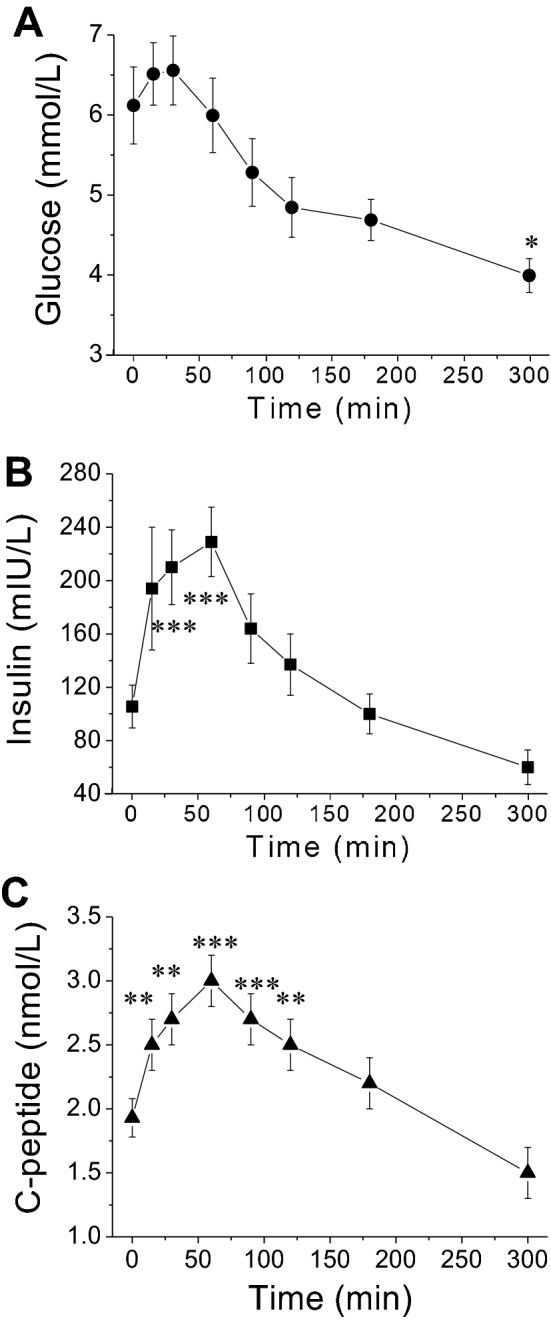
Time courses of the changes of plasma glucose, insulin and C-peptide following nasogastric meal administration at the time of 0 min. (**A**–**C)** are the concentration changes of plasma glucose, insulin and C-peptide, respectively. Values are means ± SEM (n = 16). For the plasma glucose, insulin and C-peptide responses to the MMTT, there were significant time effects (RM One Way ANOVA; **p < 0.01; ***p < 0.001; vs the baseline time 0) by Dunnett’s multiple comparisons test.

To investigate the effects of the MMTT, sixteen obese (11.2 ± 0.3 kg, Table 1) NHPs were administered with the mixed meal solution plus acetaminophen at 5 mL/kg via nasogastric tube. Plasma glucose increased from 6.1 ± 0.5 mmol/L to a maximum 6.6 ± 0.4 mmol/L (10.1 ± 4.9% increase, *p* = 0.947) at 30 min after the meal challenge (Fig. 1A). Plasma insulin and C-peptide increased (from 106 ± 16 mIU/L to 229 ± 26 mIU/L and 1.9 ± 0.2 nmol/L to 3.1 ± 0.2 nmol/L, respectively) peaking at 60 min (138 ± 29% and 63 ± 14%, respectively, *p* < 0.05 for both, Fig. 1B,C). After their peaks, all the parameters gradually declined toward their pre-MMTT levels or below pre-meal baseline levels (Fig. 1).

**Figure 2:**
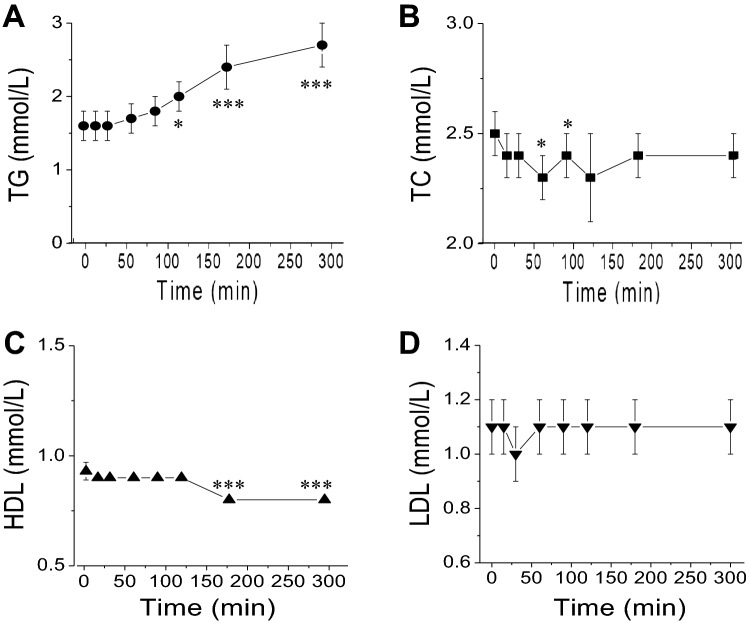
Time courses of the changes of plasma TG, TC, HDL and LDL following nasogastric meal administration at the time of 0 min. (**A**–**D)** are their concentration changes, respectively. Values are means ± SEM (n = 16). For the plasma TG and HDL responses to the MMTT, there were significant time effects (RM One Way ANOVA; *p < 0.05; ***p < 0.001; vs the baseline time 0) by Dunnett’s multiple comparisons test. For the plasma TC and LDL responses, there were no significant time effects (*p* > 0.05).

Of plasma lipids measured, only LDL, has not significantly changed after the meal challenge (Fig. 2). Plasma TG increased from 1.6 ± 0.2 mmol/L to 2.7 ± 0.2 mmol/L at 300 min (72 ± 8% increase, *p* < 0.05) (Fig. 2A) after the meal challenge. Plasma TC decreased significantly at 60 and 90 min (*p* < 0.05) and decreased to 2.33 ± 0.13 mmol/L from 2.46 ± 0.13 mmol/L at 120 min (*p* = 0.216) (Fig. 2B). Plasma HDL decreased from 1.6 ± 0.2 mmol/L to 0.81 ± 0.04 mmol/L at 300 min (19.2 ± 2.1% decrease, *p* < 0.05) (Fig. 2C) with no significant (*p* > 0.05) changes in LDL (Fig. 2D) after the meal challenge. It is not clear why the plasma TC and HDL levels significantly decreased during the MMTTs.

**Figure 3:**
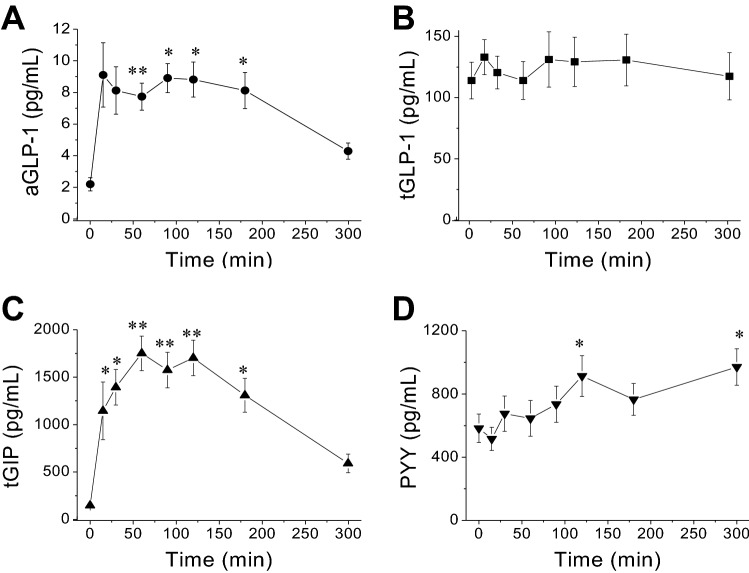
Time courses of the changes of plasma aGLP-1, tGLP-1, tGIP and PYY after nasogastric meal administration at the time of 0 min. (**A**–**D)** are their concentration changes, respectively. Values are means ± SEM (n = 16). For the plasma aGLP-1 and tGIP responses to the MMTT, there were significant time effects (RM One Way ANOVA; *p < 0.05; **p < 0.01; vs the baseline time 0) by Dunnett’s multiple comparisons test., but not tGLP-1 (*p* > 0.05).

A time course for plasma GLP-1, GIP and PYY measured from 8 animals challenged with MMTT (Fig. 3). Plasma active GLP-1 (aGLP-1) concentration increased from 2.4 ± 0.6 pg/mL to a maximum 9.2 ± 2.1 pg/mL (399 ± 117% increase, at 120 min, *p* < 0.05) after the meal administration (Fig. 3A). While, plasma total GLP-1 (tGLP-1) showed a small increase from 117.5 ± 22.2 pg/mL to 156.3 ± 40.1 pg/mL (26.7 ± 14.4% increase, *p* = 0.41) at 90 min after the meal challenge (Fig. 3B). Plasma total GIP (tGIP) increased from 151.2 ± 17.9 pg/mL to a maximum 1637.9 ± 241.7 pg/mL (increased 1276 ± 437.6%, *p* < 0.05) after the meal challenge (Fig. 3C). Plasma PYY increased slowly from 623.5 ± 168.3 pg/mL to a maximum 952.0 ± 195.1 pg/mL (increase 100.1 ± 48.5%, *p* = 0.014) and reached the highest level at 300 min after the meal administration (Fig. 3D).

**Figure 4:**
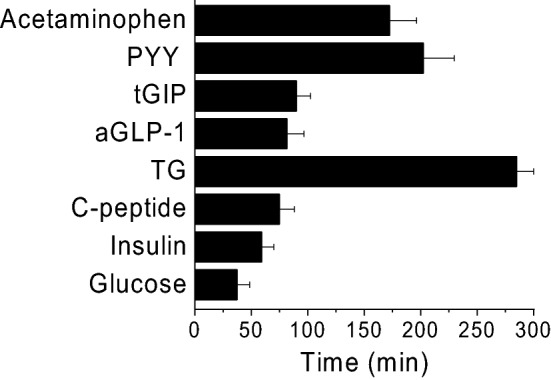
Times to reach its peak concentrations (Tmax) of plasma glucose, insulin, C-peptide, TG, aGLP-1, GIP, PYY and acetaminophen after nasogastric meal administration at the time of 0 min. Tmax was calculated according to the time reaching the peak concentration of each parameter in individual animal. Values are means ± SEM (n = 16). As TC, LDL and tGLP-1 did not show their obvious peaks during MMTTs, their Tmaxs were not calculated and presented.

Plasma levels of glucose, insulin, C-peptide, TG, aGLP-1, tGIP, and PYY reached its peak at various time points (Fig. 4). The rank order for these parameters to reach their peak plasma levels was glucose (37 ± 10 min) < insulin (59 ± 10 min) < C-peptide (75 ± 13 min) < aGLP-1 (81 ± 15 min) < tGIP (90 ± 12 min), PYY (190 ± 12 min) < TG (285 ± 15 min) (Fig. 4).

### Gastric emptying and MMTT reproducibility

Plasma acetaminophen rapidly absorbed following the oral MMTT plus acetaminophen administration in 16 monkeys. The maximum plasma acetaminophen concentration (Cmax) and the time to reach its maximum plasma concentration (Tmax) for the 1st study were Cmax = 1288.2 ± 102 ng/mL and Tmax = 195 ± 22 min (n = 16), respectively (see Fig. 4).

**Figure 5:**
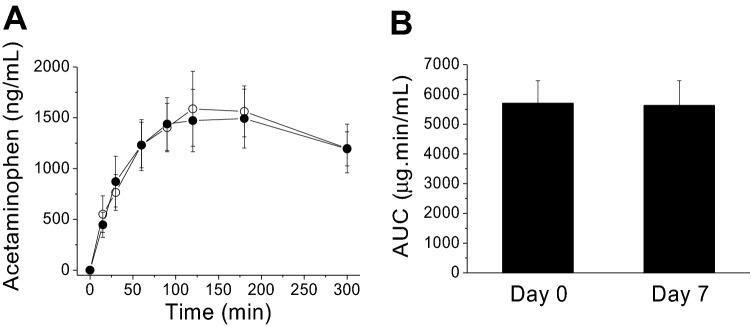
Time courses for plasma acetaminophen appearance (**A**) following a MMTT containing acetaminophen administration in two separate studies with one-week apart and comparison of the AUCs of plasma acetaminophen concentrations obtained from the two MMTT studies in one cohort of monkeys (n = 6). The patterns and time courses of plasma acetaminophen concentrations from two MMTTs were very similar. The AUCs of plasma acetaminophen concentrations showed no significant difference between two MMTTs by two-tailed Welch’s t-test (*p* > 0.05, **B**). Values are means ± SEM (n = 6). For the plasma acetaminophen responses, there was a significant time effect (*p* < 0.001) but no significant difference (*p* > 0.05) between the two MMTTs by Mixed-effects analysis followed by Sidak’s multiple comparisons test.

To evaluate reproducibility of the MMTT, a cohort of monkeys (n = 6, Table 1), underwent a second MMTT one week after the first MMTT study. The time course of plasma acetaminophen appearance was reproducible between the 1st and 2st MMTTs (Fig. 5A). The area under the curves (AUCs) of acetaminophen time courses were almost identical between the two MMTTs (Day 0: 384,232 ± 51,640.9 ug*min/mL vs Day 7: 378,474.5 ± 55,910.2 ug*min/mL, *p* = 0.941) (Fig. 5B).

The response patterns and amplitudes and the calculated total AUC_0-300 min_ of plasma glucose (Week 1: 309.2 ± 161.7 vs Week 2: 703.9 ± 147.3, *p* = 0.101), insulin (Week 1: 12,599.9 ± 8181.8 vs Week 2: 12,229.8 ± 18,591.2, *p* = 0.896), C-peptide (Week 1: 141.6 ± 51.8 vs Week 2: 165.1 ± 105.8, *p* = 0.848), and TG (Week 1: 154.7 ± 26.8 vs Week 2: 148.9.8 ± 24.8, *p* = 0.877), were also reproducible as no statistical difference observed between the two MMTTs in the 6 monkeys. Also, there were no obvious differences in plasma TC, HDL and LDL levels measured between the two MMTT studies (data are not shown). Plasma incretins were not measured during the 2st MMTT.

### Comparison of MMTT effects in hyperglycemic and normoglycemic NHPs

**Figure 6:**
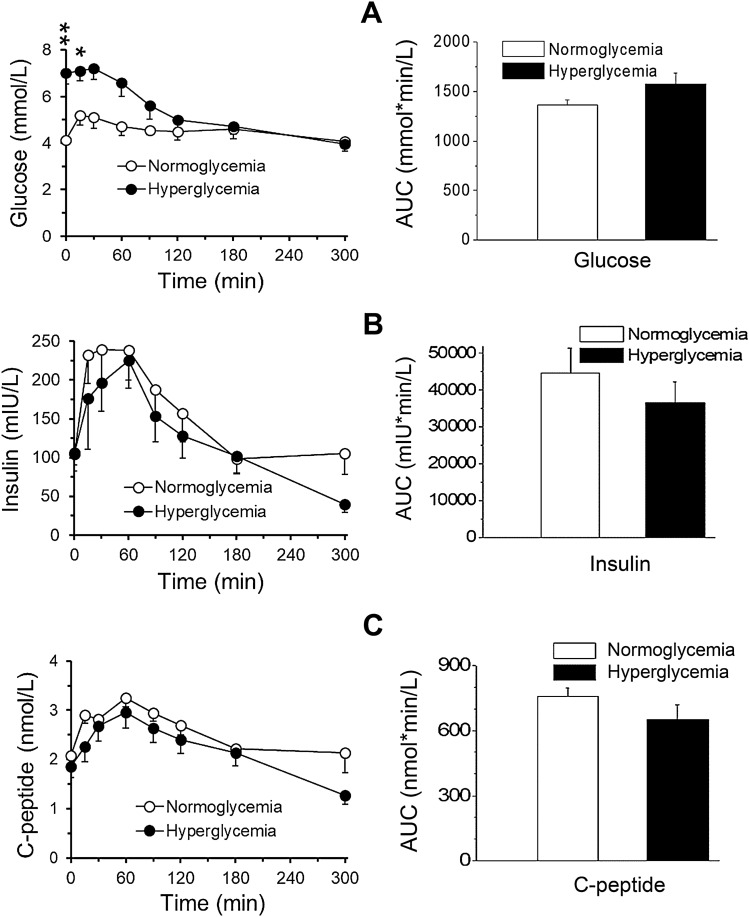

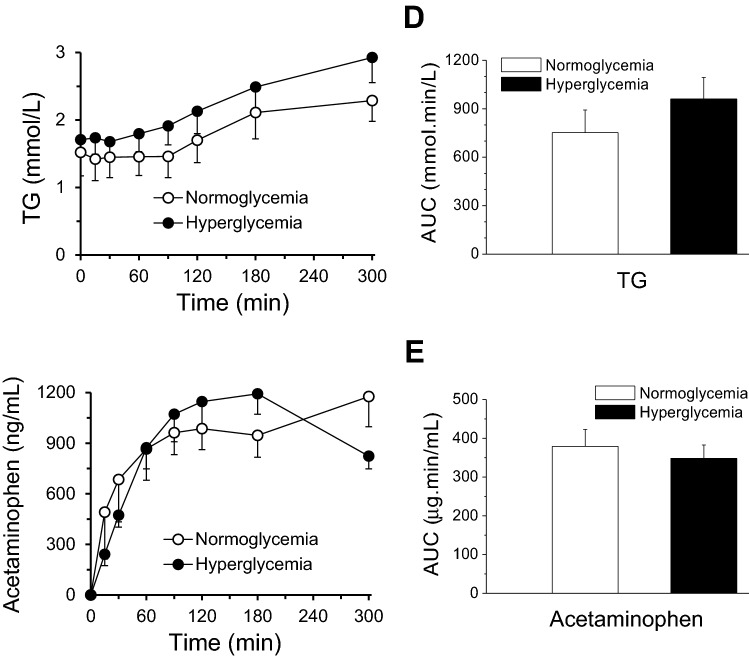
Time course for changes in plasma glucose, insulin, C-peptide, TG and acetaminophen concentrations following nasogastric meal administration at the time of 0 min (the baseline) in normoglycemic (n = 5) and hyperglycemic (n = 11) obese monkeys. (**A**–**E**) are the concentration changes (left panels) following the time after meal administration and their corresponding AUCs (right panels) of plasma glucose, insulin, C-peptide, TG and acetaminophen, respectively. These AUCs did not show significant differences (*p* > 0.05) between normoglycemic and hyperglycemic groups.

Detailed analysis of fasted baseline blood glucose and insulin levels of the first MMTT with the 16 obese monkeys (Table 1) revealed that 11 monkeys had significantly higher blood glucose levels (7.0 ± 0.5 mmol/L) than the other 5 monkeys (4.1 ± 0.2 mmol/L) (*p* < 0.01) (Fig. 6A), while fasted baseline plasma insulin and C-peptide levels were not statistically different between the hyperglycemic and normoglycemic groups (Fig. 6B,C). The baseline fasted glucose/insulin ratio was 2 folds higher in the hyperglycemic (0.1 ± 0.02) monkeys compared with the normoglycemic (0.048 ± 0.01) monkeys (*p* = 0.051). The calculated values of HOMA-IR were 19.1 ± 2.7 and 34.5 ± 9.1 for normoglycemic and hyperglycemic groups, respectively. Compared with the normoglycemic monkeys, hyperglycemic animals increased the HOMA-IR by 80%, but the difference between two groups was not statistically significant (*p* = 0.286).

We compared the responses to the MMTT challenges between the hyperglycemic (n = 11) and normoglycemic (n = 5) monkeys. Plasma glucose response to the MMTT challenge was greater in the normoglycemic relative to the hyperglycemic monkeys (at 15 min post MMTT challenge, plasma glucose increased by 25.4% *vs* 1.3% in normoglycemic *vs* hyperglycemic monkeys). Plasma glucose total AUC_0–300 min_ was also higher by 13.4% (*p* = 0.22) in the hyperglycemic monkeys (Fig. 6A), while plasma glucose net AUC_0–300 min_ was increased by 530% in normoglycemic relative to hyperglycemic monkeys (*p* < 0.001). The responses of plasma insulin and C-peptide to the MMTT administration were not markedly different except at last 300 min measurements (*p* < 0.05 for both) between the obese normoglycemic *vs* hyperglycemic monkeys. The time course and integrated total AUC of plasma TG were not significantly (*p* > 0.05) different between normo- and hyper-glycemic groups after the MMTT challenges (Fig. 6D). The changes of total AUCs of other lipid parameters (TC, HDL and LDL) were also not significantly different (*p* > 0.05) between the two groups during MMTTs (data not shown). In addition, the time course and integrated total AUC of plasma acetaminophen were not significantly (*p* = 0.255) different between normo- and hyper-glycemic groups after the MMTT challenge (Fig. 6E). Based on the results from the power simulations analysis for plasma acetaminophen from the 16 monkeys used, 5 monkeys per group will be required to achieve statistical 0.8 power with effect size of 20%. While 14 monkeys per group will be required to achieve statistical 0.8 power with effect size of 10%.

**Figure 7:**
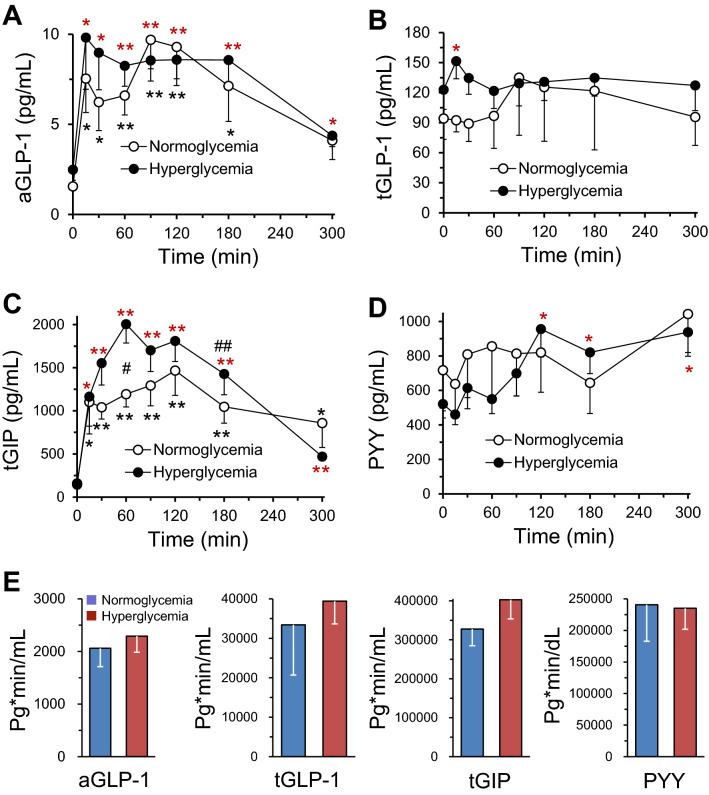
Time course for changes in plasma aGLP-1 (**A**), tGLP-1 (**B**), tGIP (**C**) and PYY (**D**) concentrations following nasogastric meal administration immediately after the time of 0 min (baseline) in normoglycemic (n = 5) and hyperglycemic (n = 11) monkeys. (**E**) Shows the corresponding total AUCs of plasma aGLP-1 in (**A**), tGLP-1 in (**B**), tGIP in (**C**) and PYY in (**D**) of the MMTTs. These AUCs did not show significant differences (*p* > 0.05) between normo- (* or **) and hyper-( or ) glycemic groups. *, *p* < 0.05 or **, *p* < 0.01; vs the self-baseline at time 0 min. ^#^*p* < 0.05 or ^##^*p* < 0.01; vs the normoglycemic group.

Baseline plasma active GLP-1 (Normoglycemic: 1.56 ± 0.21 pg/mL vs hyperglycemic: 2.49 ± 0.59 pg/mL, *p* = 0.164), tGLP-1 (Normoglycemic: 94.24 pg/mL ± 20.81 pg/mL vs hyperglycemic: 122.96 ± 19.57 pg/mL, *p* = 0.336), GIP (Normoglycemic: 157.56 ± 21.14 pg/mL vs hyperglycemic: 143.71 ± 22.86 pg/mL, *p* = 0.664) and PYY (Normoglycemic: 717.1 ± 235.4 pg/mL vs hyperglycemic: 521.3 ± 80.97 pg/mL, *p* = 0.467) were not significantly different between the normo- and hyper-glycemic groups (*p* > 0.05, Fig. 7, data-points at time 0). Compared with the baselines at time 0, the levels of plasma aGLP-1 were significantly increased during the MMTTs in both normo- and hyper-glycemic groups, except 300 min time point (*p* > 0.05) in normoglycemic animals (Fig. 7A), but the increases at any time points differed insignificantly (*p* > 0.05) between the two groups. Plasma tGLP-1 significantly increased only at 15 min time point in the hyperglycemic animals (Fig. 7B). Also at 15 min time point, plasma tGLP-1 was higher (*p* = 0.05) in the hyperglycemic animals than in normoglycemic obese monkeys. The levels of plasma tGIP were significantly increased in both normo- and hyper-glycemic groups (Fig. 7C) during the MMTTs. The increases in plasma tGIP at the time points of 60 and 180 min after administration of the mixed meals were significantly more in the hyperglycemic monkeys than in those normoglycemic ones (Fig. 7C). The levels of plasma PYY during the MMTTs were significantly increased only at the time points of 120, 180 and 300 min in hyperglycemic animals (Fig. 7D). Interestingly, the values of the AUCs for aGLP-1, tGLP-1, tGIP and PYY after the MMTT challenges were not significantly different between the normo- and hyper-glycemic groups (Fig. 7E).

## Discussion

The current studies were undertaken to characterize the metabolic responses and assess gastric emptying to a MMTT in spontaneously obese cynomolgus monkeys. The reproducibility of the MMTT was robust for the metabolic biomarkers and plasma acetaminophen appearance. The nasogastric feeding tube, which was used in our experimental set up, assured that the complete and homogeneous meal/acetaminophen mixture was in the stomach at the start of the experiment. After administering the meal/acetaminophen, appearance of acetaminophen plasma was seen at the first (15 min) measurement, indicative for a relatively rapid small intestine absorption of acetaminophen in obese monkeys. Plasma acetaminophen AUCs were similar between the two MMTT studies, supporting the reproducibility and reliability of the procedure used in obese monkeys. In human with T2DM, a standardized MMTT reproducibility has been reported to be moderately dependent on parameter (glucose, insulin or C-peptide) and size of population^37^.

The gastric emptying rate profiles after oral administration of acetaminophen in cynomolgus monkeys and humans are similar^38^. Gastric emptying kinetics is often closely monitored during the development of a new drug candidates to find out its pharmacokinetics and efficacy after dosing. In healthy subjects, the acetaminophen absorption test produced results comparable to those of liquid gastric emptying scintigraphy test^39^. The rate of orally administered acetaminophen absorption accurately reflects the rate of gastric emptying^25,26^. Acetaminophen can thus be considered as a suitable tool compound for evaluating the effect of the drug on gastric emptying. It is well established that the rate of gastric emptying is determined by various factors, including the size, density and content of the meal, such as high- or low-calorie^40^ or high or low fatty acid contents^41^. Gastric emptying and acetaminophen absorption are also affected by various drugs, i.e. opioids^27^ and GLP-1agonists used for treatment of diabetes and obesity^42^. Acetaminophen provides a convenient, safe, and relatively inexpensive method for the measurement of liquid-phase gastric emptying and small intestinal absorption in human patients. Our data support that the acetaminophen method is a repeatable method for evaluating gastric emptying in the NHPs.

Meal ingestion induces postprandial metabolic responses, including changes in glucose, insulin, gut hormones (incretins) and lipids^43^*.* In the current study the MMTT plus acetaminophen caused insignificant rise in blood glucose at 30 min measurement accompanied with significant increases in plasma insulin, C-peptide, aGLP-1, tGIP and TG after the meal administration. Baseline levels of overnight fasted glucose and insulin, time courses and AUCs of glucose and insulin for obese hyperglycemic and normoglycemic groups are consistent with reported data in cynomolgus monkey model of spontaneously developed diabetes^32,34^. It has been reported that fasted blood glucose > 125 mg/dL (> 7 mmol/L) represents progression from prediabetes to overt diabetes, and this could be hastened in NHPs by feeding the monkeys a high fat high glucose or high-sugar diets^44^. These diabetic monkeys have higher plasma insulin levels relative to their controls. In our study, the obese hyperglycemic monkeys had fasted glucose ~ 7 mmol/L and obese normoglycemic monkeys had fasted blood glucose of ~ 4 mmol/L (~ 72 mg/dL). There was no difference in fasting insulin between the obese hyper- and normo-glycemic monkeys, possibly due to obesity and hyperglycemia developing with age as opposed to diet. In addition, all the animals increased their HOMA-IR due to obesity. There was no statistical difference in HOMA-IR between the hyper- and normo-glycemic monkeys, even the hyperglycemic animals showed 80% increase in HOMA-IR. The reported values of HOMA-IR in normal monkeys were in a range of 2.5 to 8.4 and significantly increased in obese, pre-diabetic or diabetic cynomolgus monkeys^45,46^ and also in rhesus monkeys with the metabolic syndrome^47^. The increase in HOMA-IR was more obvious in pre-diabetic monkeys than in obese or diabetic monkeys^45^.

In the present study, fasting plasma glucose, but not insulin or C-peptide, was significantly higher in the hyperglycemic monkeys compared to normoglycemic group. The higher baseline and net AUC of glucose in hyperglycemic group relative to normoglycemic group may indicate more glucose intolerance in the hyperglycemic group. This is consistent with a previous report in cynomolgus monkeys with naturally occurring diabetes^32,34^. Both insulin and C-peptide trended lower than basal levels in obese hyperglycemic than normoglycemic group at the 300 min blood sample collection, while glucose levels at 300 min were not different between the two groups. Maintaining the blood glucose to baseline levels after MMTT in controls and lack of this regulation in hyperglycemic group may be due to the disturbance in the mechanisms regulating glucose in metabolic disease. It has been reported that blood glucose reduced by 14.7% at 240 min relative to baseline in age induced-prediabetic, but not in type 2 diabetic cynomolgus monkeys fed on high fat diet^48^.

The incretin hormones, GIP and GLP-1are released from intestinal K- and L-cells, respectively when nutrients pass through the intestine^49^. Circulating GLP-1 and GIP exert glucose lowering effects by directly binding to their respective G-protein coupled receptors on pancreatic β-cells to regulate insulin secretion in a glucose-dependent manner to maintain glucose homeostasis^50,51^.

In the present study, the administration of a mixed meal rapidly increased levels of GIP and active GLP-1 in the obese NHPs. The rapid increase in GIP after the meal is consistent with previous report in naturally occurring diabetes in cynomolgus monkeys challenged with a meal^34^. However, unlike the significant increase in active GLP-1 observed in our study, others^34^ reported no change after a meal in plasma GLP-1 levels in naturally occurring diabetic cynomolgus monkeys. In our study, both active and total GLP-1 were measured, the change in total GLP-1 was very small, similar to what was reported previously^34^, which may suggest that they may have measured total and not active GLP-1.

The incretins, GIP and active GLP-1 responses to the meal administration were comparable between the normoglycemic and hyperglycemic animals. This is consistent with Sun et al.^34^, who reported similar changes for GIP and GLP-1 AUCs following MMTT in naturally occurring diabetic cynomolgus monkeys relative to their controls. The increase in plasma active GLP-1 in our study resembles changes reported in type 2 diabetics after MMTT, while the rapid increase in plasma GIP in our study resembles the changes reported in pre-diabetic and diabetic patients following a MMTT challenge^52^. Our GLP-1 results are consistent with the previous report which shows that the GLP-1 levels and the AUC of GLP-1 following MMTT did not differ among non-diabetic individuals with morbid obesity, diabetic individuals with mild obesity and healthy controls^53^. However, in T2DM patients the plasma concentrations of GLP-1 are diminished, but GLP-1 stimulation of insulin secretion is preserved. In contrast to GLP-1, the plasma concentrations of GIP are normal in T2DM patients, but the effect of GIP on insulin secretion is reduced due to defective expression and downregulation of GIP receptors in pancreatic β-cells^50,51^. Our result also differs from what has been reported in type 2 diabetic patients, where plasma GIP changes were reduced in the diabetic than healthy controls during MMTT^12^. Some discrepancies between our monkey data and the results reported in humans, such as aGLP-1 and GIP, may result from the differences in species, longevities and disease stages, as well as other experimental conditions between monkeys and patients, which can complicate the outcomes for comparison. In addition, diabetic patients are often under antidiabetic treatment and our experimental monkeys are not under any therapeutic treatments. Therefore, extra caution is needed if using our monkey data interprets physiology or pathophysiology in humans. PYY, a master regulator of food intake, is released from the intestinal L-cells, mainly in the ileum, colon, and rectum, acts via G-protein coupled NPY receptors. Despite PYY and GLP-1 are co-localized in intestinal L-cells, however, unlike the rapid increase in GLP-1, PYY gradually increased after a meal, with a peak at 3 h or longer in obese monkeys in our study. In humans with T2DM, but not in prediabetic patients, circulating PYY levels were reported to be low during fasting, but rapidly increased after a meal with a peak after 1 to 2 h, remaining elevated for several hours^54^. Like GLP-1 and GIP, the AUC of PYY was not different between obese hyperglycemic and normoglycemic monkeys in our study. Interestingly, PYY changes were related to changes of plasma TG over time. It is unclear how these two parameters are connected, although it is known that the macronutrient content (fat, carbohydrate and protein) of the meal plays an important role in incretin secretion^50,51^. In addition, it is reported that dietary fat and protein stimulates PYY secretion, while fat and carbohydrates stimulate GIP and GLP-1 secretion^55–57^.

We recognize that the interpretation of our data may be limited by the small animal number of the normoglycemic group (n = 5), which may not have been sufficiently powered to detect differences between the two groups. In addition, compared with the normoglycemic group, the plasma glucose levels of the animals in the hyperglycemic group were significantly higher, but they still showed very good preservation of β-cell function based on the baseline plasma insulin and C-peptide levels and their levels during MMTT. The hyperglycemic animals still showed insulin-sensitivity in regulation of post-meal blood glucose, because both the baseline and secreted insulin and C-peptide levels were similar between hyperglycemic and normoglycemic groups. The increases in blood glucose levels during MMTT were minimal and similar in both groups. These results suggest that the hyperglycemic animals might not have severe metabolic disturbances other than hyperglycemia as all the monkeys enrolled in this study were obese with increased HOMA-IR.

In summary, out data demonstrate that the MMTT can provide a reliable and convenient method to test metabolic functions in NHPs. The present results suggest that the MMTT is applicable for examining incretins and glucose homeostasis with additional valuable information which the intravenous or oral glucose tolerance test may miss. Thus, the MMTT can be an excellent method to test β-cell function in NHPs for obesity and diabetes research. The addition of acetaminophen provides as a reliable and reproducible test for routine preclinical evaluation of gastric emptying in NHPs as needed.

## Data Availability

All the materials and relevant raw data supporting our findings can be found in Tables and Figures in the manuscript and are freely available to readers or scientists wishing to use them for non-commercial purposes.

## Abbreviations

AAALAC: Association for Assessment and Accreditation of Laboratory Animal Care
ANOVA: Analysis of variance
AUC: Area under the curve
DPP IV: Dipeptidyl peptidase IV
DEXA: Dual-energy X-ray absorptiometry
ELISA: Enzyme-linked immunosorbent assay
GLP-1: Glucagon-like peptide-1
GIP: Gastric inhibitory polypeptide
GST: Glucagon stimulation test
HDL: High density lipoprotein
HOMA-IR: Homeostatic Model Assessment of Insulin Resistance
HRP: Horseradish Peroxidase
IACUC: Institutional animal care and use committee
i.m.: Intramuscular injection
LDL: Low density lipoprotein
LLOD: Lower limit of detection
MMTT: Mixed meal tolerance test
NHP: Nonhuman primate
OD: Optical density
PYY: Peptide YY
SEM: Standard error of the mean
T2DM: Type 2 diabetes mellitus
TC: Total cholesterol
TG: Triglyceride
